# Jumping the Gun: Mapping Neural Correlates of Waiting Impulsivity and Relevance Across Alcohol Misuse

**DOI:** 10.1016/j.biopsych.2015.06.009

**Published:** 2016-03-15

**Authors:** Laurel S. Morris, Prantik Kundu, Kwangyeol Baek, Michael A. Irvine, Daisy J. Mechelmans, Jonathan Wood, Neil A. Harrison, Trevor W. Robbins, Edward T. Bullmore, Valerie Voon

**Affiliations:** a Department of Psychology, University of Cambridge; b Behavioural and Clinical Neuroscience Institute, University of Cambridge; c Department of Psychiatry, University of Cambridge, Addenbrooke’s Hospital, Cambridge, United Kingdom; d Section on Advanced Functional Neuroimaging, Translational and Molecular Imaging Institution, Icahn School of Medicine at Mt. Sinai, New York, New York; e Cambridgeshire and Peterborough National Health Service Foundation Trust, Cambridge; f Department of Psychiatry, Brighton and Sussex Medical School, Brighton; g National Institute for Health Research Biomedical Research Council, University of Cambridge, United Kingdom

**Keywords:** Addiction, Binge drinking, Connectivity, Impulsivity, Machine learning, Subthalamic nucleus

## Abstract

**Background:**

Why do we jump the gun or speak out of turn? Waiting impulsivity has a preclinical basis as a predictor for the development of addiction. Here, we mapped the intrinsic neural correlates of waiting and dissociated it from stopping, both fundamental mechanisms of behavioral control.

**Methods:**

We used a recently developed translational task to assess premature responding and assess response inhibition using the stop signal task. We mapped the neural correlates in 55 healthy volunteers using a novel multi-echo resting-state functional magnetic resonance imaging sequence and analysis, which robustly boosts signal-to-noise ratio. We further assessed 32 young binge drinkers and 36 abstinent subjects with alcohol use disorders.

**Results:**

Connectivity of limbic and motor cortical and striatal nodes mapped onto a mesial-lateral axis of the subthalamic nucleus. Waiting impulsivity was associated with lower connectivity of the subthalamic nucleus with ventral striatum and subgenual cingulate, regions similarly implicated in rodent lesion studies. This network was dissociable from fast reactive stopping involving hyperdirect connections of the pre-supplementary area and subthalamic nucleus. We further showed that binge drinkers, like those with alcohol use disorders, had elevated premature responding and emphasized the relevance of this subthalamic network across alcohol misuse. Using machine learning techniques we showed that subthalamic connectivity differentiates binge drinkers and individuals with alcohol use disorders from healthy volunteers.

**Conclusions:**

We highlight the translational and clinical relevance of dissociable functional systems of cortical, striatal, and hyperdirect connections with the subthalamic nucleus in modulating waiting and stopping and their importance across dimensions of alcohol misuse.

Why do we jump the gun, speak out of turn, or run a red light? Waiting and stopping are fundamental mechanisms of behavioral control. The tendency toward rapid unplanned reactions without adequate forethought broadly defines impulsivity (1). Converging preclinical and clinical evidence suggests impulsivity to be a heterogeneous construct of differing subtypes, with distinct but overlapping neural substrates (1, 2, 3). The propensity for impulsivity varies across individuals and may contribute to everyday suboptimal behaviors such as overeating and poor financial management. This ability to control impulsive behavior is impaired across a range of neuropsychiatric disorders including disorders of addiction.

Here, we explore this phenomenon in three studies. The first characterizes the neural correlates of waiting impulsivity, or anticipatory premature responding before target onset, in healthy volunteers (HV). The second and third studies assess waiting impulsivity in binge drinkers (BD) and examine the same neural correlates across social drinkers, binge drinkers, and alcohol use disorders (AUDs), respectively. This form of impulsivity is well characterized in rodents through the 5-choice serial reaction time task (5-CSRT) (4) and has important preclinical evidence supporting its role as a predictor for the development of disorders of addiction (5, 6, 7). High premorbid premature responding in rodents predicts greater nicotine use (6) and greater addiction-like behavior to cocaine (5). Mice acutely exposed to alcohol (8) in the early stages of alcohol abstinence (9) and with greater preference for alcohol (10) all exhibit enhanced premature responding. Using a novel translational task, the human 4-choice serial reaction time task (4-CSRT), which was designed with high fidelity to the rodent 5-CSRT (11, 12), we have previously shown that premature responding is enhanced across individuals with alcohol and methamphetamine dependence and is elevated in smokers and recreational cannabis users (11). While the apparent transdiagnostic relevance of premature responding is clear, the underlying neural correlates in humans are yet to be elucidated.

Work in rodents has provided evidence for candidate neural regions for waiting impulsivity. Lesion studies have identified a specific network underlying premature responding implicating the nucleus accumbens (or human ventral striatum), infralimbic cortex (probably equivalent to human subgenual anterior cingulate), and subthalamic nucleus (STN). For example, nucleus accumbens lesions attenuate amphetamine-induced increases in premature responding (13). Highly impulsive rodents have lower D_2/3_ nucleus accumbens receptor availability (14) and reduced nucleus accumbens core gray matter density (15). Furthermore, lesions of the rodent infralimbic cortex and the STN also enhance premature responding (16, 17, 18, 19).

Special interest falls on the STN, a major relay structure within the indirect inhibitory pathway of striatal circuitry, which also receives hyperdirect projections directly from cortical regions (20, 21). This rich convergence of cortical inputs implicates the STN as a crucial mediator of more complex control of motor and cognitive function. In humans, high-frequency deep brain stimulation (DBS) targeting the STN, in which high-frequency stimulation is delivered to targets of interest, thus modulating networks, is increasingly used for the symptomatic management of refractory obsessive-compulsive disorder (22) and is already established as highly effective for symptomatic management of Parkinson’s disease. DBS provides insight into the STNs role and can serve as a model for STN neuromodulation and impulsivity. In rodents, increasing amplitudes of STN DBS at high frequencies increases premature responding (23). STN DBS can modulate several subtypes of impulsivity (24, 25, 26) characterized predominantly by reactive stopping with tasks involving explicit signals or enhanced responding to irrelevant stimuli or conflict. The current study focuses on premature responding or, more precisely, the capacity to wait before responding to a cue predicting reward (12).

In the first study, we examined the neural correlates of waiting impulsivity in healthy volunteers using the novel 4-CSRT and further differentiated that network from another underlying a well-characterized form of motor impulsivity, measured using the stop-signal task. With the stop-signal task, both rodent and human studies showed that action cancellation of a prepotent ongoing motor response (motor response inhibition) is dissociable from premature responding (11, 27). Converging studies on stopping behaviors implicate hyperdirect connections to the STN, including from the pre-supplementary motor area (pre-SMA) and right inferior frontal cortex, as well as the indirect pathway output of the dorsomedial striatum (caudate) (3, 28, 29). To examine the intrinsic neural correlates of waiting and stopping, we used resting-state functional magnetic resonance imaging (fMRI) sequence and multi-echo independent components analysis, which has been shown to have up to fourfold enhancements in signal-to-noise ratio relative to single-echo fMRI scans (30), thus enabling high-fidelity assessment of small subcortical structures like STN in terms of parcellation and interregional connectivity. Based on preclinical data, we hypothesized that greater waiting impulsivity would be associated with decreased connectivity of the STN with the ventral striatum and subgenual cingulate cortex. To dissociate waiting impulsivity from motor response inhibition, we further hypothesized that impaired response inhibition as measured with the stop-signal task would be associated with lower hyperdirect connectivity of the pre-SMA and right inferior frontal cortex with STN.

We further extended a translational focus investigating premature responding in binge drinkers in the second study and examined the currently implicated neural correlates of premature responding across social drinkers, binge drinkers, and those with alcohol use disorders in the third study. We have previously shown that AUDs have elevated premature responding, tested using the 4-CSRT (11). As young adult binge drinkers are at elevated risk for developing AUD (31), we hypothesized in the second study that binge drinkers, similar to those with AUD, would have elevated waiting impulsivity. In the third study, we examined the neural correlates of waiting impulsivity, expecting that both binge drinkers and those with AUD would have decreased intrinsic connectivity of the described network. On an exploratory basis, using machine learning classification, we assessed the extent to which STN network connectivity would allow for classification of pathological drinkers from healthy volunteers.

## Methods and Materials

### Participants

Subjects were scanned with a resting-state sequence. Healthy volunteers completed two behavioral tasks outside the scanner (offline). Supplement 1 includes all subject characteristics. Baseline functional connectivity of the STN with cortical and striatal regions was assessed in 66 HV. The neural correlates of waiting and motor impulsivity were examined in 55 HV, who completed both behavioral tasks along with imaging.

The recruitment strategy for HV and pathological drinkers (BD and AUD) has been previously reported (11). For the second study, we examined behavioral impulsivity in 32 BD compared with 64 age- and gender-matched HV (19 HV overlapped with the 55 HV who completed both behavioral tasks along with imaging). In the third study, STN connectivity maps of 36 abstinent subjects with AUD and 32 BD who underwent scanning were compared with matched HV. Age-matched HV were separately tested for each patient group (for AUD, 34 HVs; for BD, 32 HVs). Finally, data from a proportion of the HV (social drinkers, *n* = 38) and binge drinkers (*n* = 32) who completed the scanning and the Alcohol Use Disorders Test (AUDIT) (32) were examined.

The diagnostic and screening criteria are reported in Supplement 1. Of the AUD group, we have previously reported elevated premature responding (11), elevated delay discounting and impaired motor response inhibition (33), elevated risk seeking to likely but small rewards (34), and a shift from habitual to goal-directed learning strategies with abstinence (35).

### Tasks

#### Premature Responding

**Figure 1:**
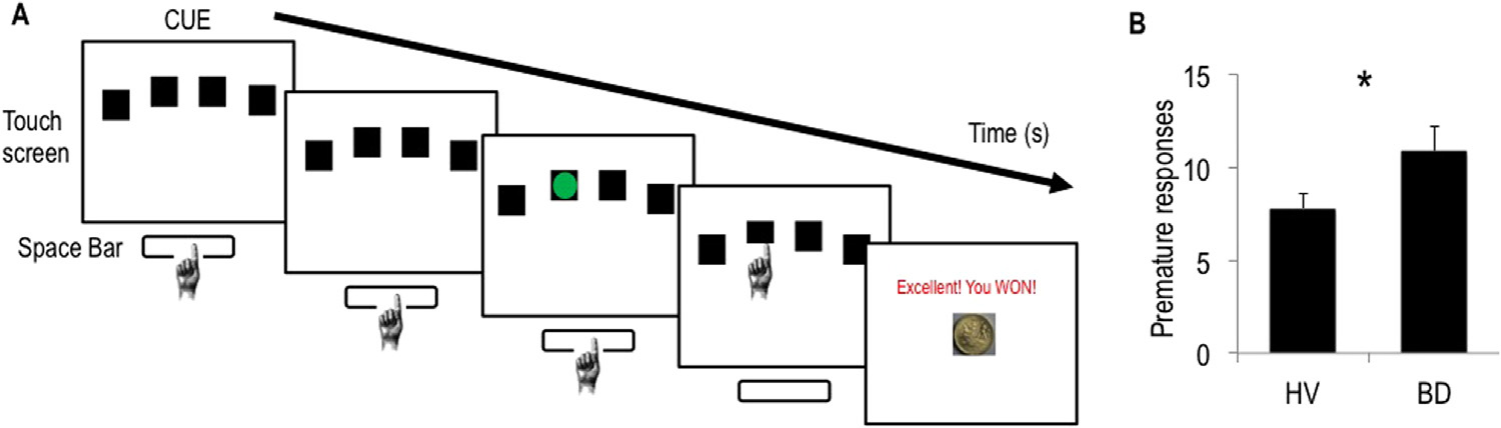
Waiting impulsivity in binge drinkers. **(A)** The 4-choice serial reaction time task. The cue onset prompted subjects to hold down the space bar. A green circle target appeared, to which subjects responded by releasing the space bar and touching the box on the touch screen in which the target appeared. Finally, monetary feedback was displayed. Premature responding was defined as release of the space bar before target onset. **(B)** Number of premature responses of healthy volunteers (HV) and binge drinkers (BD); error bars are standard error of the mean; *indicates *p* < .05.

The 4-CSRT task (Figure 1) was developed based on the rodent 5-CSRT. When four boxes appeared on the screen, subjects held down the space bar on the keyboard with their dominant index finger, indicating the cue onset time. After a specified period (cue-target interval), a green circle target appeared briefly and randomly in one of the four boxes. Subjects released the space bar and touched the box in which the target appeared. Premature responding was defined as early release of the space bar before target onset. Supplement 1 includes further task details.

#### Stop Signal Task

The stop signal task (36) is described in Supplement 1.

### Resting State Data Acquisition

To examine the underlying neural networks associated with our measures, we analyzed blood oxygen level-dependent (BOLD) fMRI data during rest. We employed a novel multi-echo planar sequence and independent components analysis in which BOLD signals were identified as independent components having linear echo time dependent signal change and non-BOLD signals were identified as echo time independent components (30). Resting-state fMRI data were acquired for 10 minutes with eyes open, fixating on a cross on a screen. Supplement 1 includes acquisition parameters.

### Data Analysis

Multi-echo independent component analysis (ME-ICAv2.5 beta6; http://afni.nimh.nih.gov) (30) was used for analysis and de-noising of the multi-echo resting-state fMRI data. Functional connectivity analysis was performed using a region of interest (ROI)-driven approach with CONN-fMRI Functional Connectivity toolbox (37) for SPM8 (Wellcome Trust Center for Neuroimaging, Institute of Neurology, University College London, London, United Kingdom; http://www.fil.ion.ucl.ac.uk/spm/software/spm8/). Supplement 1 includes further descriptions of multi-echo independent components analysis and CONN-fMRI processing.

#### Underlying Connectivity of STN with Motor, Cognitive, and Limbic Regions

ROI-to-voxel whole-brain connectivity maps were computed for cortical (pre-SMA and SMA) and striatal (ventral striatum and posterior putamen) seeds and functional connectivity was restricted to the anatomical STN. The pattern of connectivity of each seed within the STN was examined.

#### Study 1: Neural Correlates of Waiting Impulsivity

ROI-to-ROI correlation coefficients for carefully defined ROIs based on strong a priori hypotheses (STN with ventral striatum and subgenual cingulate cortex) for each HV were obtained. Pearson’s correlation was computed between the averaged BOLD time courses within the STN ROI and the averaged BOLD time courses within the ventral striatal or subgenual cingulate cortex ROI. This was also done for STN with pre-SMA, right inferior frontal cortex, and dorsal caudate for the stop-signal task. These coefficients were then correlated with the behavioral measures obtained from the tasks described, as well as AUDIT scores.

#### Study 2: Behavioral Waiting Impulsivity in Binge Drinkers

To compare behavioral measures between groups, BD subjects were compared with age- and gender-matched HV. Stop-signal reaction time (SSRT) was assessed using multivariate analyses. The Mann-Whitney *U-*test (premature responding) was used for outcomes that were not normally distributed; *p* < .05 was considered significant for a priori hypothesized analyses.

#### Study 3: Subthalamic Nucleus Connectivity in Pathological Drinking

For the estimation of differences between pathological drinkers and HVs, ROI-to-voxel whole-brain connectivity maps were computed for STN. These connectivity maps were entered into full factorial general linear models to compare whole-brain connectivity between groups. Whole-brain voxel-wise group comparisons were performed using cluster extent threshold correction at 15 voxels at *p* < .001 whole-brain uncorrected, which corrects for multiple comparisons at *p* < .05 assuming an individual voxel type I error of *p* = .01 (38). Secondarily, for strong a priori hypothesized regions, STN connectivity was compared between groups using small volume corrections (SVC). A familywise error (FWE) threshold of *p* < .05 within the SVC was considered significant for these tests. Supplement 1 describes anatomical ROI generation.

For exploratory supervised machine learning classification analysis, we used support vector machine (SVM) using Pattern Recognition for Neuroimaging Toolbox for SPM8 (39), further described in Supplement 1. The same STN ROI-to-voxel connectivity maps were entered as input data. Significance was assigned at *p* < .05 for the combined pathological drinking groups compared with HVs. Each drinking group was then compared with their own age-matched HV in two separate SVM analyses with the same parameters.

## Results

### Intrinsic Cortical and Striatal Connectivity With STN

**Figure 2:**
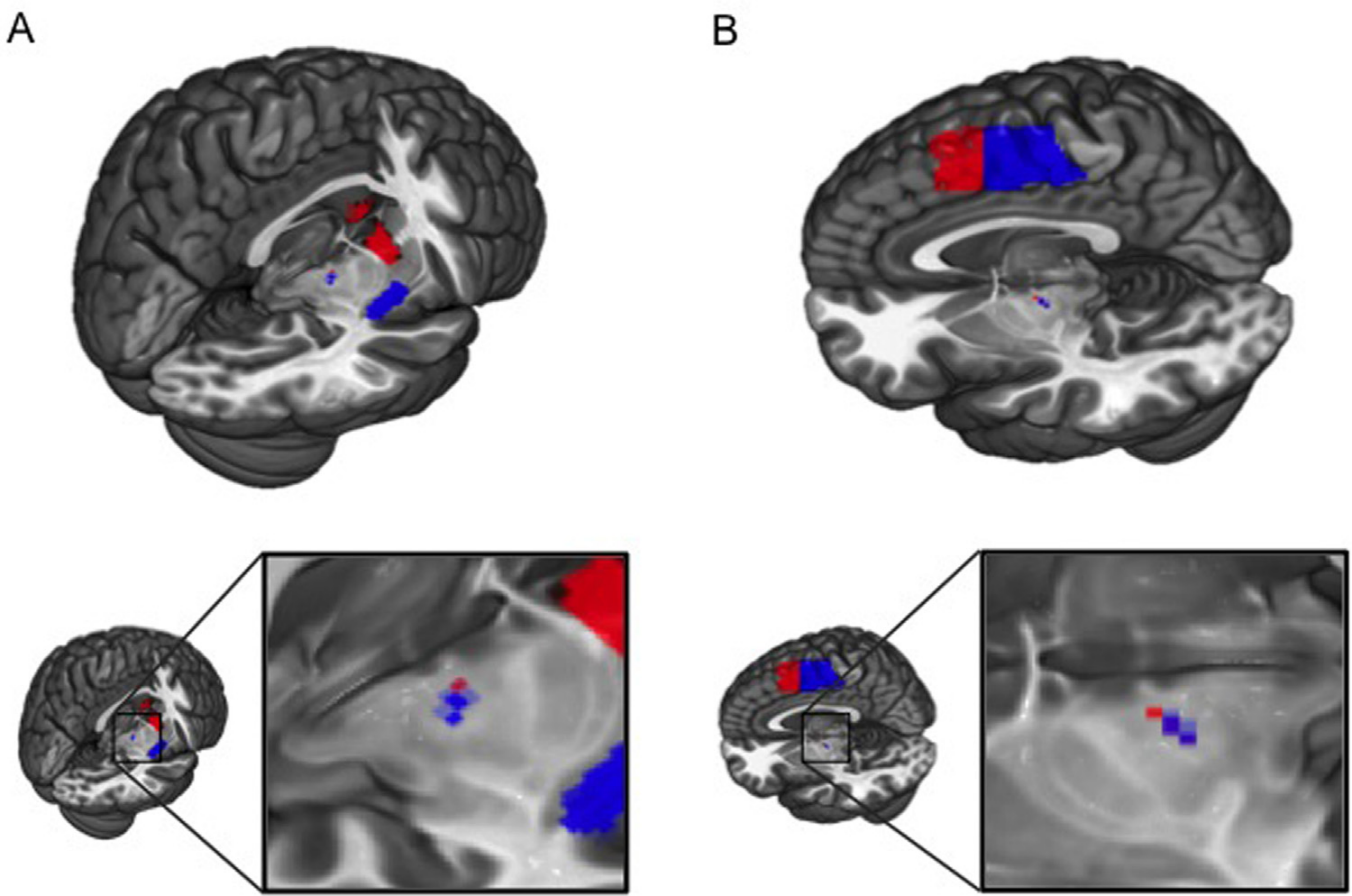
Intrinsic limbic and motor connectivity of the subthalamic nucleus. Distinct intrinsic connectivity of motor and limbic regions with subthalamic nucleus (STN) computed via seed-to-voxel correlation analysis. Analyses were restricted to STN small volume correction at familywise error threshold (*p* < .05 for ventral striatum and *p* < .001 for others). **(A)** Activity of ventral striatum (red) and posterior putamen (blue) correlated with medial and dorsolateral STN, respectively. **(B)** Supplementary motor area (blue) and pre-supplementary motor area (red) also correlated with distinct subdivisions of STN. See Table 1.

We first mapped intrinsic connectivity of cortical and striatal regions onto STN in 66 HV (Table S1 in Supplement 1 illustrates subject characteristics). Activity within each seed region significantly correlated with STN activity (statistics are reported in Table 1). Functional connectivity between motor and limbic regions and the STN were dissociable on a lateral-medial axis. Seeds in the SMA and pre-SMA were correlated with peak STN correlations in the lateral portion with pre-SMA being more dorsolateral (Figure 2; Table 1). Posterior putamen seed was correlated with the posterolateral, motor portion of the STN, while ventral striatum seed was exclusively correlated with the medial, limbic tip (Figure 2; Table 1).

### Study 1: Neural Correlates of Waiting Impulsivity

To characterize the correlates of waiting impulsivity in HV, we scanned 55 HV (Table S1 in Supplement 1) with a multi-echo fMRI resting-state sequence and examined measures from separate behavioral testing using the 4-CSRT task (Figure 1) (11) and the stop-signal task (36). We quantified functional connectivity between ROIs by calculating the Pearson correlation coefficient of time series from a priori hypothesized region pairs (henceforth referred to as connectivity). These connectivity values were then correlated with behavioral measures, with age as a covariate of no interest. The following results are reported for bilateral ROIs unless stated otherwise.

**Figure 3:**
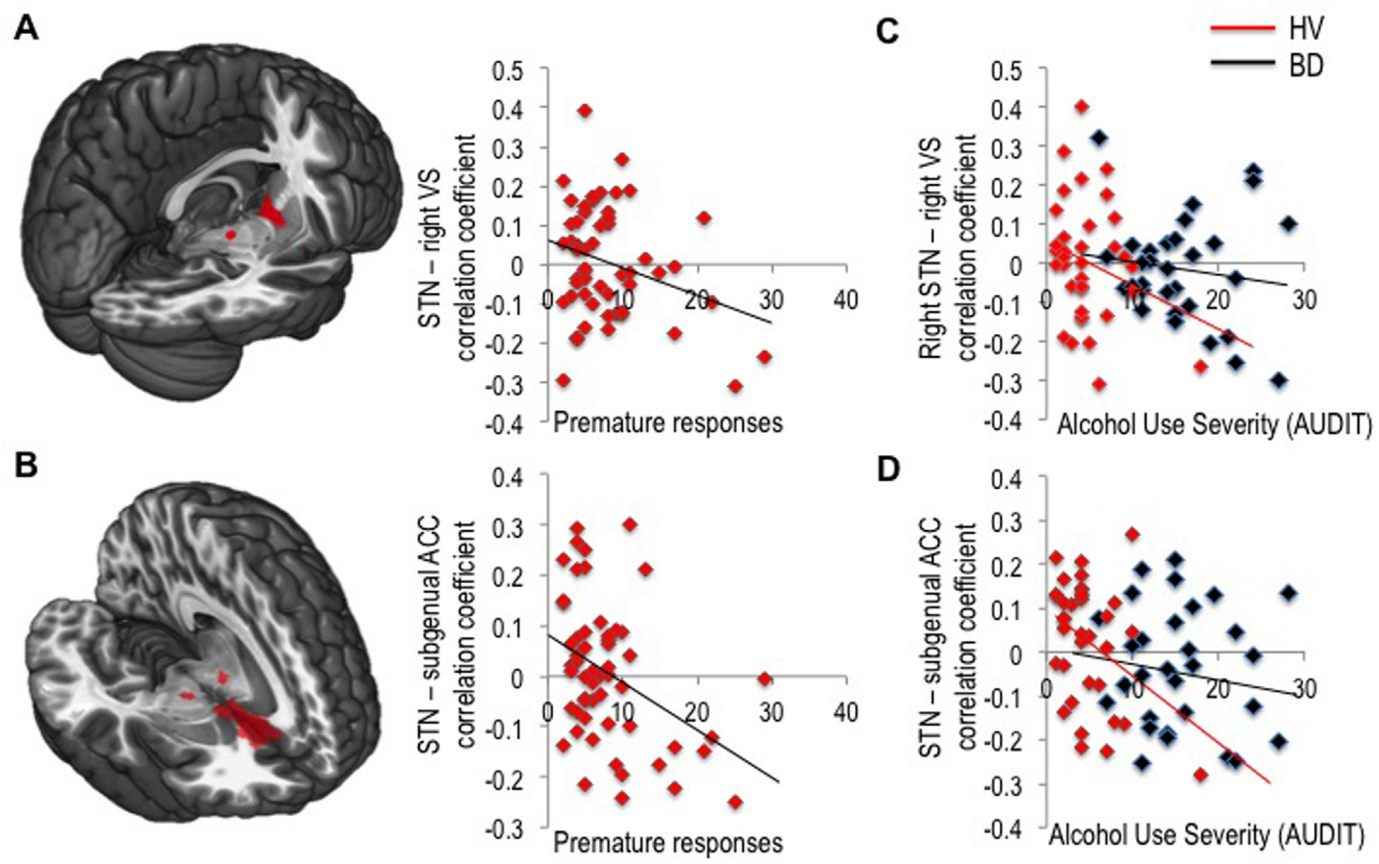
Neural correlates of waiting impulsivity and alcohol use severity. Correlation coefficients were computed between regions of interest (ROI) as a marker of their functional connectivity. Functional connectivity between subthalamic nucleus (STN) and ventral striatal (VS) seed regions **(A)** and STN and subgenual anterior cingulate cortex (ACC) **(B)** was correlated with premature responses in healthy volunteers (HV). The seed regions for ROI-to-ROI analyses are shown overlaid on Montreal Neurological Institute 152 template image. Connectivity between the same regions correlated with the Alcohol Use Disorders Test (AUDIT) scores in healthy volunteers together with binge drinkers (BD) **(C, D)**.

Greater premature responding was negatively correlated with connectivity between STN and right ventral striatum (*r* = −.286, *p* = .034; Figure 3A) and, more strikingly, between STN and subgenual cingulate cortex (*r* = −.391, *p* = .003; Figure 3B) thus confirming our primary hypotheses. We repeated the analysis with SSRT as a covariate of no interest, which did not affect the results. Similarly, SSRT was not correlated with connectivity between STN and ventral striatum or subgenual cingulate.

**Figure 4:**
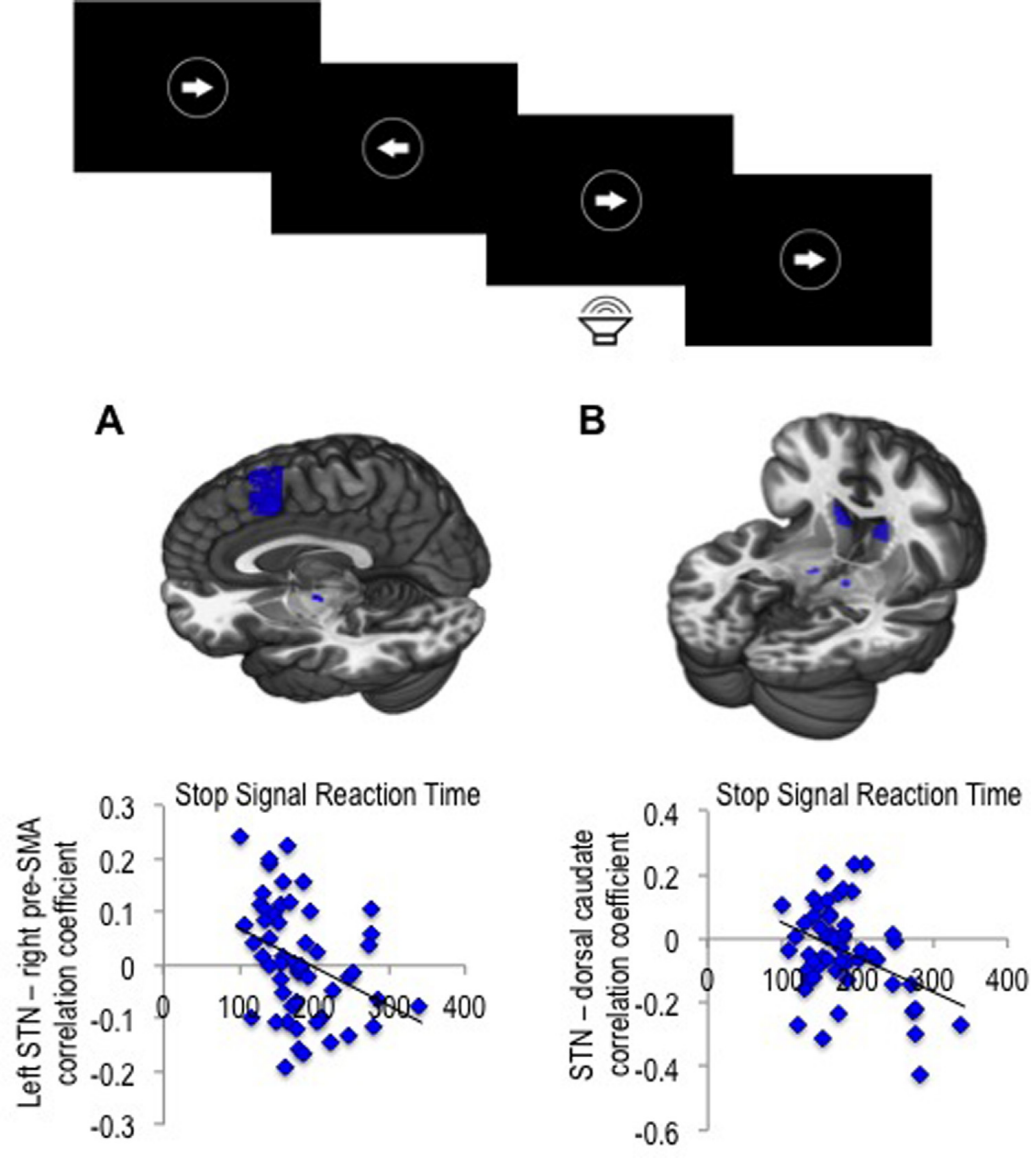
Neural correlates of motor response inhibition. Stop signal task (top). Functional connectivity (measured as correlation coefficients) between subthalamic nucleus (STN) and pre-supplementary motor area (pre-SMA) **(A)** and dorsal caudate **(B)** was correlated with stop-signal reaction time in healthy volunteers. The regions of interest (ROIs) for ROI-to-ROI analyses are shown overlaid on Montreal Neurological Institute 152 template image.

We then explored the relationship with the stop-signal task. The stop-signal task data of one participant were removed as an outlier for all analyses using this measure (Go reaction time score >3 SD above group mean). SSRT was negatively correlated with connectivity between left STN and right pre-SMA connectivity (*r* = −.350, *p* = .010) along with STN and dorsal caudate connectivity (*r* = −.338, *p* = .014) (Figure 4). There was no significant correlation between SSRT and connectivity between STN and right inferior frontal cortex. These hyperdirect and striatal connections with STN were also not significantly correlated with premature responding.

### Study 2: Behavioral Waiting Impulsivity in Binge Drinkers

We have previously shown that AUD subjects compared with HV have elevated premature responding (11), greater motor response inhibition with higher SSRT (33), and greater delay discounting. Here, we compared 32 BD subjects with 64 age- and gender-matched HV (Table S1 in Supplement 1 contains subject characteristics). Premature responding in the 4-CSRT was greater in BD (10.86 [SD 7.21]) compared with HV (7.81 [SD 6.77]; *p* = .041) (Figure 1) with no differences in SSRT (HV: 165.65 [SD 57.46]; BD: 160.92 [SD 27.37]; *p* = .320). Supplement 1 includes additional data.

### Study 3: Subthalamic Nucleus Connectivity in Pathological Drinking

To establish whether STN connectivity differs with clinically relevant alcohol misuse, we examined STN connectivity of 36 abstinent subjects with AUD (Table S1 in Supplement 1 includes subject characteristics; reported in mean [SD]: weeks abstinent 15.78 [17.13], range 2–52; years heavy use 13.29 [8.31]; units/day 29.44 [15.31]; on the following medications, acamprosate 2 and disulfiram 1) and 32 young adult subjects with BD who underwent scanning compared with age- and gender-matched HV (for AUD, 34 HVs; for BD, 32 HVs). Smoking status of current/ex-smokers/never was AUD, 21/3/8; BD, 12/5/13; HV for BD 4/3/21; and HV for AUD, 5/7/19.

**Figure 5:**
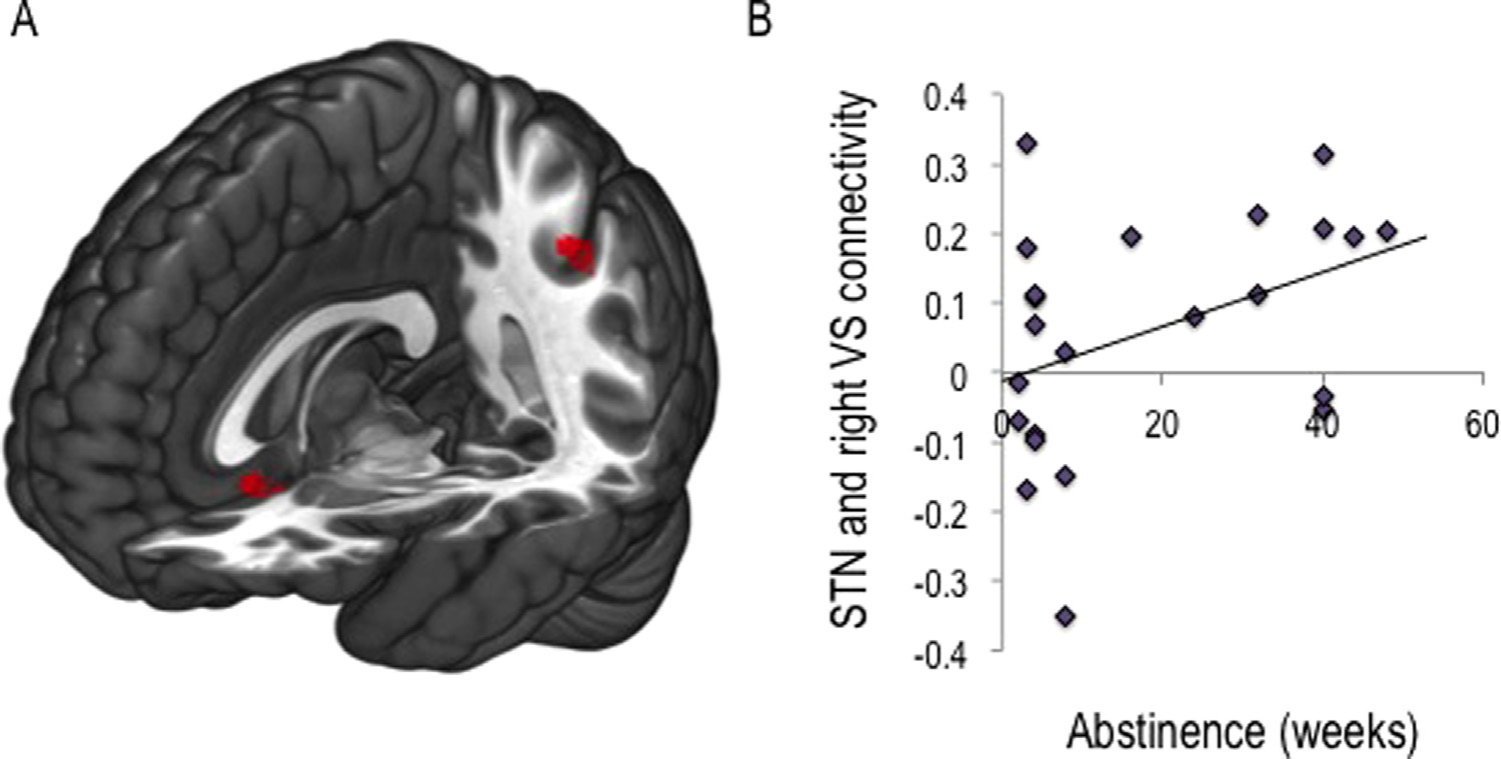
Subthalamic nucleus connectivity in binge drinkers and alcohol use disorders. **(A)** Independent samples *t* test to compare region of interest to whole-brain voxel connectivity maps for subthalamic nucleus (STN) between groups revealed reduced connectivity of the STN with both the subgenual cingulate cortex and the inferior parietal cortex (cluster-extent threshold analysis *p* < .05) compared with age- matched healthy volunteers. **(B)** A trend toward a positive correlation (*p* = .058) was observed between weeks abstinent and connectivity between STN and right ventral striatum (VS) in individuals with alcohol use disorder.

STN ROI-to-whole-brain voxel connectivity maps were entered into independent sample *t* tests to compare groups. Group comparisons were performed using cluster extent threshold correction, calculated at 15 voxels at *p* < .001 whole brain uncorrected, correcting for multiple comparisons at *p* < .05 assuming an individual voxel type I error of *p* = .01 (38). Cluster-extent threshold analysis revealed that both AUD and BD had reduced STN connectivity with subgenual cingulate cortex (peak reported in Montreal Neurological Institute coordinates xyz = −45 −41 42 mm; cluster size *=* 45; *Z* = 4.32; *p* = .036; Figure 5A) and inferior parietal cortex (xyz = 1 19 −10 mm; cluster size = 58; *Z* = 4.83; *p* = .019) compared with HVs.

We further examined specific group differences in STN connectivity with ventral striatum and subgenual cingulate (reported as SVC FWE corrected for these a priori hypothesized regions). As expected, compared with HVs, both BD and AUD had reduced connectivity of STN with subgenual cingulate cortex (*Z* = 4.32, *p* = .002). Both BD and AUD also exhibited reduced connectivity of STN with ventral striatum (*Z* = 4.10, *p* = .006). As the combined group was significant overall, the groups were then separately compared with age-matched HVs. Relative to their matched HVs, AUD subjects had reduced STN and subgenual cingulate cortex connectivity (*Z* = 3.47, *p* = .040) and BD had reduced ventral striatum (*Z* = 3.97, *p* = .010) and subgenual cingulate cortex (*Z* = 3.96, *p* = .008) connectivity. We secondarily examined the currently implicated neural correlates of SSRT: there were no group differences in STN connectivity with pre-SMA or caudate (*p*>.05). Since smoking affects premature responding (11), we added smoking status (current or never/ex-smoker) as a covariate of no interest in the main group difference analysis. The main findings of reduced STN connectivity with ventral striatum and subgenual cingulate in pathological drinkers remained significant. Connectivity of the STN with ventral striatum or subgenual cingulate cortex was also not different between current versus never and ex-smokers in the HV group (*p* < .05). However, STN connectivity with pre-SMA was higher in never smokers compared with current and ex-smokers (SVC FWE, peak coordinates, −8 21 44, *Z* = 3.57, *p* = .046).

### Exploratory Machine Learning Analysis

On an exploratory basis, supervised machine learning methods (support vector machine) were applied to STN seed ROI-to-whole-brain voxel connectivity maps to determine whether signal patterns in the data could be used to classify between groups. Correct classification was achieved for all pathological drinking groups versus HVs with a significant balanced accuracy of 59.8% (*p* = .039) (see Supplement 1 for model weights map). AUD and BD were then separately compared with their own age-matched HV: correct classification was achieved for BD with significant balanced accuracy of 71.9% (*p* = .026). SVM analyses of AUD showed an elevated balanced accuracy of 64.7% that was not significant (*p* > .05).

### Subthalamic Nucleus Connectivity as a Function of Alcohol Use Severity and Abstinence

We further examined the relationship between the neural network associated with waiting impulsivity and severity of alcohol use in a proportion of the healthy volunteers (social drinkers, *n* = 38) and binge drinkers (*n* = 32) (Table S1 in Supplement 1). Across both groups, AUDIT scores negatively correlated with connectivity between STN and subgenual cingulate cortex (*r* = −.391, *p* = .001; Figure 3C) and with a trend correlation with connectivity between right STN and right ventral striatum (*r* = −.236, *p* = .052). To determine whether this represented an underlying biomarker, we examined the HV group alone. In HVs, AUDIT scores negatively correlated with connectivity between STN and subgenual cingulate cortex (*r* = −.421, *p* = .010) but not STN and ventral striatum (*r* = −.267, *p* = .11). In AUD, there was a positive correlation trend between the number of weeks abstinent and STN connectivity with right ventral striatum (*r* = .411, *p* = .058; Figure 5B) and no correlations with units per day or total units consumed.

## Discussion

This study assessed the neural correlates of waiting and stopping. Greater premature responding in humans using the novel 4-CSRT task was associated with decreased intrinsic connectivity of bilateral STN with bilateral subgenual cingulate and right ventral striatum. Our findings provide translational evidence in humans for a similar network implicated in rodents. These findings are dissociable from motor response inhibition or action cancellation as captured by SSRT, which was associated with lower connectivity between hyperdirect projections of the right pre-SMA and left STN along with dorsal caudate and STN connectivity. We used a novel multi-echo resting-state fMRI sequence and analysis allowing for marked improvements in signal-to-noise ratio. Limbic and motor corticostriatal circuitry mapped to a mesial-lateral STN axis. Together, our findings implicate dissociable parallel functional corticostriatal and hyperdirect systems in modulating waiting and stopping.

We highlight the translational potential of these findings in alcohol misuse. Young adult BD subjects at greater risk for subsequent alcohol use problems showed enhanced premature responding relative to matched healthy volunteers. This adds to our previous report of enhanced premature responding using the 4-CSRT in AUD subjects relative to healthy volunteers (11) and supports a previous finding of enhanced premature responding using a motor task in BD subjects (10). Furthermore, in healthy social drinkers, the degree of alcohol severity correlated negatively with connectivity between the bilateral STN and subgenual cingulate. We highlight this as a potential early clinical marker that we further explored with predictive algorithmic modeling. Using machine-learning classification, STN connectivity differentiated BD and AUD from social drinkers (healthy volunteers). This may be driven by the decreased connectivity in BD and AUD between the STN with the subgenual cingulate and inferior parietal cortex. Compared with age-matched healthy volunteers, both AUD and BD had decreased connectivity between bilateral STN and subgenual cingulate, not attributed to smoking status. Nicotine use appeared to influence the connectivity between the STN and pre-SMA in healthy volunteers, suggesting a potential influence on the neural correlates of reactive stopping. However, as the sample size was small, we emphasize these findings are preliminary. While STN and whole-brain connectivity classified pathological drinking groups versus healthy volunteers, it was not sufficient to classify between AUD alone and healthy volunteers. The balanced accuracy in AUD, while elevated, failed to reach significance. This may be related to abstinence in this group, as we find a trend toward stronger connectivity between STN and ventral striatum with longer abstinence. This may also explain why we did not find group differences of STN and ventral striatal connectivity for AUD. Together, the findings suggest that the neural correlates of premature responding, particularly connectivity between the STN and subgenual cingulate, may be endophenotypic markers of alcohol misuse but that STN and ventral striatal connectivity may act as a neuroadaptive marker.

Interestingly and at odds with evidence of impairments in response inhibition in patients with AUD (40), we did not find group differences in the neural correlates of stopping (STN with pre-SMA or dorsal caudate). However, reports of SSRT impairments are inconsistent, including both impaired response inhibition (41) and no differences compared with healthy volunteers (42). Furthermore, group differences in neural correlates of response inhibition focus on dorsolateral prefrontal cortex (42), right inferior frontal cortex (36), and frontal cortex connectivity with putamen (43), which we did not examine. Thus, while the characterization of common underlying deficits can be demonstrated across overlapping disorders, additional behavioral and neurobiological constructs specific to an individual disorder must be simultaneously evaluated.

These findings dovetail with preclinical observations of enhanced premature responding in a gambling task in rodents with STN lesions (17, 18, 19). Increasing amplitudes of STN DBS at high frequencies increase premature responding in rodents (23). In human studies, STN DBS in patients with Parkinson’s disease increases several subtypes of impulsivity, including impaired motor response inhibition (25), faster reaction time to conflict (24), and enhanced early errors in the Simon task (26). The Simon task has been divided into early and late responses in which errors in the early phase represent early capture to irrelevant stimuli (i.e., arrow in opposite direction) and errors in the late phase represent impairments in response inhibition. This early responding to irrelevant stimuli is associated with increased pre-SMA activity and is believed to be unrelated to inhibitory processes (44). Transient suppression of the pre-SMA with transcranial magnetic stimulation is also associated with a decrease in early responses in the Simon task, particularly in reward conditions (45). There may be similarities in the relationship between premature responding and these early stimulus-invoked responses (12); however, as we did not observe a relationship between premature responding and pre-SMA and STN connectivity, this remains to be clarified. While much evidence implicates STN DBS in enhancing impulsivity, a study of Parkinson’s disease demonstrated that STN DBS was associated with a decrease in impulse control disorders (46), suggesting a more complex relationship with impulsivity.

The STN is critically implicated in response inhibition via the indirect and hyperdirect pathways. STN lesions in rodents are associated with a generalized impairment in response inhibition with greater errors in the stop-signal task but no specific prolongation of SSRT (47). Our findings of separable neural networks underlying premature responding and action cancellation (SSRT) converge with rodent and human studies in demonstrating that the two measures are unrelated (11, 27). In rodents, action restraint as measured using commission errors in go/no-go type tasks is similarly unrelated to premature responding (27, 48). The STN is believed to influence response suppression by inhibition of thalamocortical pathways via both reactive (in response to an internal or external cue) and proactive (preparatory inhibition) models (24, 25). Our findings suggest that premature responding is likely dissociable from fast reactive stopping as mediated via the fast hyperdirect pre-SMA–STN projection. Alternatively, a slower tonic inhibition suppressing automatic responses to irrelevant stimuli including high conflict or prepotent responses may still be relevant in premature responding.

While the current study did not directly address the neural correlates of waiting impulsivity at the time of the behavior (in-scanner testing), we highlight intrinsic neural correlates. Furthermore, as we did not include causality analyses, we did not clarify directionality of connectivity findings.

We highlight dissociable frontal/striatal and hyperdirect neural networks involving STN underlying waiting and stopping. These findings have important mechanistic implications and are relevant to DBS targeting the STN for Parkinson’s disease and obsessive-compulsive disorder. We further highlight a dimensional approach to the neural correlates underlying premature responding across alcohol misuse consistent with the current trend toward dimensional psychiatry (49).

## Figures and Tables

**Table 1:** Statistics of Cortical and Striatal Seed Connectivity With Subthalamic Nucleus

	*p*(FWE-corr)	*Z*	x	y	z
Supplementary Motor Area	<.001	6.15	−10	−16	−5
Pre–Supplementary Motor Area	<.001	5.47	−13	−11	−3
Posterior Putamen	<.001	6.29	13	−16	−5
Ventral Striatum	.035	2.72	8	−11	−5

*p*(FWE-corr) is small volume corrected familywise error *p* value, using STN for small volume correction.

FWE, familywise error; STN, subthalamic nucleus; xyz, peak voxel coordinates; *Z*, *Z*-score.
