# Supplementary material for: Jumping the Gun: Mapping Neural Correlates of Waiting Impulsivity and Relevance Across Alcohol Misuse
Source: Biol Psychiatry. 2016 Mar 15;79(6):499–507. doi: 10.1016/j.biopsych.2015.06.009 (PMC4764648; doi:10.1016/j.biopsych.2015.06.009)
Supplement: Supplementary file 1 — Supplementary Material [file mmc1.pdf]

Jumping the Gun: Mapping Neural Correlates of Waiting Impulsivity and Relevance Across Alcohol Misuse

Supplemental Information

Supplemental Methods & Materials

Diagnostic and Screening Criteria

Psychiatric disorders were screened with the Mini International Neuropsychiatric Interview. Participants were excluded if they had a current major depression or another major psychiatric disorder including substance addiction, major medical illness, or were taking psychotropic medication. National Adult Reading Test was used to assess IQ. Participants completed the Beck Depression Inventory (1). Participants were reimbursed for their time and written informed consent was obtained. The study was approved by the University of Cambridge Research Ethics Committee.

Binge-drinking criteria were based on the National Institute on Alcoholism and Alcohol Abuse diagnostic criteria (2) and included rapid consumption of greater than 8 or 6 units (for males and females, respectively) within 2 hours at least once a week and had been 'drunk' each time for the previous 3 months. Primary AUD diagnoses were confirmed by a psychiatrist (VV) using the Diagnostic and Statistical Manual of Mental Disorders, Version IV (DSM IV-TR) criteria for substance dependence (3).

4-CSRT

Subjects were seated in front of a touch screen (a Paceblade Tablet personal computer; Paceblade Technology, Amersfoort, the Netherlands). Baseline blocks without monetary feedback were used to individualize monetary feedback amounts for subsequent blocks based on the individual's mean fastest reaction time and standard deviation. The 4 test blocks with

monetary feedback were optimized to increase premature responding and varied by target duration, variability of the cue-target interval and the presence of distractors. Accurate and timely responses were followed by individualized reward magnitude outcomes depending on the speed of responding. The primary outcome measure was the premature release of the space bar prior to target onset. The task lasted 20 minutes and was programmed in Visual Basic with Visual Studio 2005. The premature responding task comprised of two baseline blocks and four test blocks. The baseline blocks were used to individualize feedback according to the individual's reaction time (RT) and encourage individuals to respond faster. Each baseline block had 20 trials with the final 10 trials used to calculate mean RT and standard deviation (SD) for this calculation. Baseline Block 1 occurred at the start of the trial with the mean RT used for Test Block 1. Baseline Block 2 occurred at the end of Test Block 1 with the mean RT from both baseline blocks used for Test Blocks 2 to 4. The subjects were told to respond as quickly as possible during the baseline blocks with "Keep going" appearing on the screen as feedback. There were 4 test blocks with monetary feedback (40 trials per block). Subjects were instructed to respond as quickly as possible, that they would earn money for their responses, that it was more important to be fast rather than accurate and that they would not lose money if they were inaccurate.

The relationship between baseline block mean RT, SD and test block feedback were as follows: Very fast accurate responses: For very fast accurate responses in which RT during a trial in the test blocks was less than -0.5 SD of the baseline RT, the response was followed by the text "YOU WIN!! EXCELLENT!!" along with a £1 image. If subjects won £1 in three sequential trials, the feedback increased to £2. Fast accurate responses: For accurate responses in which test RT was between -0.5 SD and +0.5 SD of the baseline RT, the response was followed by the text "Very good. Keep going." along with a 50 pence image. Test RTs that were accurate and between +0.5 SD and +1.5 SD of the baseline RT were

followed by the text “Good. Keep going.” along with a 10 pence image. Slow accurate responses: Slow but accurate responses in which trial RTs were greater than +1.5 SD of the baseline RT were penalized and followed by the text “YOU LOSE!! TOO LATE!! HURRY UP!!” and an image of -£1 with a red X over the coin. No response: If no responses were registered, the feedback was “TOO LATE!! GO FASTER!!” with an image -£1 with a red X. Premature response or incorrect responses: Neither premature responses (responding prior to target onset) nor incorrect responses (touching the incorrect box) were penalized. Following a premature response, subjects were required to touch the screen to complete the trial, which was followed by the text “Keep going”. An incorrect response was followed by the text “Keep going”.

Stop Signal Task

Response inhibition was tested using the stop signal task (CANTAB, Cambridge Neuropsychological Test Battery) (Figure 3) (4). Subjects were presented with a series of Go stimuli in the form of a left or right arrows and instructed to respond as quickly as possible by pressing the respective button on the two button response box. On a subset of the trials, the stimulus is followed 250 ms later (stop signal delay, SSD) by a stop signal tone. The SSD varied by 50 ms depending on performance, maintaining a staircase throughout the task resulting in approximately 50% successful and 50% unsuccessful stop trials. The key outcome measure was the stop signal reaction time (SSRT). The SSRT is based on Logan’s model of a competition between the stop and go response.

Resting State Data Acquisition

Data were acquired with a Siemens 3T Tim Trio scanner using a 32-channel head coil at the Wolfson Brain Imaging Centre at the University of Cambridge. Anatomical images were

acquired using a T1-weighted magnetization prepared rapid gradient echo (MPRAGE) sequence (176 x 240 field of view (FOV); 1-mm in-plane resolution; inversion time, 1100 ms). All participants underwent a resting state fMRI scan of 10 minutes. Functional images were acquired with a multi-echo echo planar imaging sequence with online reconstruction (repetition time, 2.47 s; flip angle, 78°; matrix size 64 x 64; in-plane resolution, 3.75 mm; FOV, 240 mm; 32 oblique slices, alternating slice acquisition slice thickness 3.75 mm with 10% gap; iPAT factor, 3; bandwidth = 1,698 Hz/pixel; echo time (TE) = 12, 28, 44 and 60 ms).

Multi Echo Independent Components Analysis

Multi echo independent components analysis initially decomposes multi-echo fMRI data into independent components using FastICA. Then, independent components are categorized as BOLD or non-BOLD based on their weightings measured by kappa and rho values, respectively (5). BOLD signal has percent signal changes that are linearly dependent on TE, a characteristic of the T2* decay. TE dependence of BOLD signal is measured using the pseudo-*F*-statistic, kappa, with components that scale strongly with TE having high kappa scores (5). Non-BOLD components are identified by TE independence measured by the pseudo-*F*-statistic, rho. By removing non-BOLD components (by projection), data are denoised for motion, physiological and scanner artefacts in a robust manner based on physical principles (6). Each individual's denoised echo planar images are coregistered to each individual's MPRAGE and normalized to the Montreal Neurological Institute (MNI) template with coordinates reported in MNI space.

Supervised Machine Learning

For classification analysis, we use supervised machine learning methods that automatically extract information from the data, remaining sensitive to subtle spatial differences. We used a support vector machine (SVM), Pattern Recognition for Neuroimaging Toolbox (PRoNTo) for SPM (7), which initially uses example training data that have previously been classified (i.e., patient or healthy volunteer) to identify an optimum boundary that distinguishes the training data into the 2 groups. This is followed by a testing phase in which the computed boundary is used to predict which group the new data belongs to in a blind manner. Finally its performance in doing this is evaluated. All patients and matched HV's were entered as two classes into one SVM analysis with the STN region of interest (ROI)-to-voxel whole brain connectivity maps as inputs. Cross validation of leave one subject out was used. This method uses data from all subjects except one from each group to train the classifier. The two data that were left out are then used to test the ability of the machine to classify between "new" data. This is repeated for all subjects allowing an unbiased estimate of generalizability. Statistical significance of classification was tested using permutation testing with 1000 permutations with random assignment of group class to input image. We direct the reader to an excellent review by (8) for further details of this method.

CONN-fMRI Functional Connectivity Toolbox: Processing

Spatial smoothing was conducted with a Gaussian kernel (full width half maximum = 6 mm). The time course for each voxel was temporally band-pass filtered ($0.008 < f < 0.09$ Hz). Each individual's anatomical scan was segmented into gray matter, white matter and cerebrospinal fluid (CSF). Significant principal components of the signals from white matter and CSF were removed.

Region of Interest Generation

Our analyses focused on *a priori* regions of interest for each cognitive measure. We used the anatomically defined STN ROI provided by WFU PickAtlas SPM toolbox (9) as the STN seed. This seed has a similar center of mass to a previous study focusing on the STN, which used a sphere with 3 mm radius centered on the coordinates x y z = 10, -14, -4 mm for the right STN which was selected based on an fMRI task study on stopping ability (10). This sphere had the same central coordinates as our STN ROI from WFU PickAtlas. We further confirmed our STN seeds using anatomical tracings of R2* and magnetization transfer anatomical sequences (11) manually traced for 16 healthy volunteers by two researchers. The centers of mass for the manually traced STN (0.5, -13.5, -6.7 mm) and the STN ROI from WFU PickAtlas (0.6, -13.6, -5.6 mm) were similar. We used a ventral striatal (VS) anatomical ROI, as previously in other studies (12) which was hand drawn using MRIcro based on the definition of VS by (13). The subgenual ACC was based on Brodmann's area 25 from WFU PickAtlas. The putamen ROI was obtained from the Automated Anatomical Labelling (AAL) atlas. The posterior putamen was separated from anterior putamen using a vertical line passing through the anterior commissure.

For stop signal task analyses we also examined posterior putamen, pre-SMA and right inferior frontal cortex (IFC) (10). The putamen ROI was obtained from the AAL atlas (14). The posterior putamen was separated from anterior putamen using a vertical line passing through the anterior commissure. The posterior border of the pre-SMA is typically defined as a vertical line through the anterior commissure, the anterior border defined as a vertical line passing through the genu of the corpus callosum and the inferior border being the superior border of the cingulate cortex (15). By respecting the boundaries, we created a preSMA seed. For the right IFC seed we maintained the inferior frontal sulcus as the superior boundary, the precentral gyrus as the posterior boundary and the rostral extent of the inferior frontal sulcus

as the anterior boundary (16,17). We restricted the region to that including the frontal areas falling within a 30 mm radius sphere centered on coordinates +48, 18, 8 (18).

Supplemental Results

Behavioral Results in Binge Drinkers

Alcohol Use Disorders Test scores were higher in binge drinkers (HV 4.63 (SD 3.95); BD: 15.70 (SD 5.41), $t = -9.57$, $p < 0.0001$). Alcohol intake per week in units was: HV: 4.13 (SD 2.53); BD: 15.81 (SD 5.23), $t = 12.11$, $p < 0.0001$). There were no differences in motivation index (BD: 0.17 (SD 0.15); HV: 0.15 (SD 0.14); $p = 0.489$). No differences were found for BD subjects in SSRT (HV: 168.10 (SD 55.79); BD: 170.39 (SD 19.75); $t = 0.216$, $p = 0.830$).

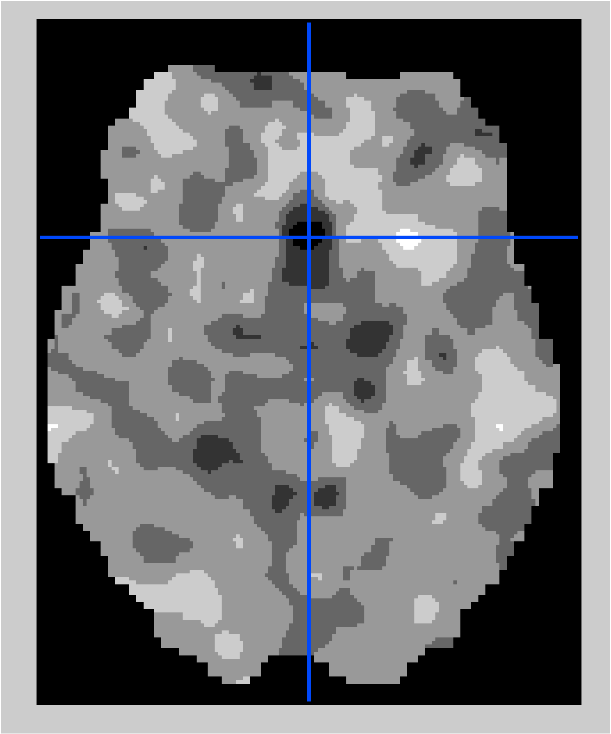

Figure S1. Model weights obtained with support vector machine with STN to whole brain connectivity map data. Image created by Pattern Recognition for Neuroimaging Toolbox (PRoNTo) comprising the weight vectors for the linear classifier. Note that no threshold has been applied to this image.

Table S1. Subject characteristics.

	Group	M/F	Age (SD)	Verbal IQ (SD)	BDI (SD)	AUDIT (SD)
Intrinsic cortical and striatal connectivity with STN	HV	33/33	40.0 (13.3)	114.3 (9.5)	8.6 (8.4)	
Study 1: Neural correlates of waiting impulsivity	HV	28/27	38.9 (13.4)	115.6 (8.9)	8.5 (8)	
Study 2: Behavioral waiting impulsivity in binge drinkers	HV	36/28	23.1 (4.2)	115.9 (6.9)	5.1 (4.4)	4.5 (3.5)
	BD	18/14	22.1 (3.3)	116.5 (5.3)	7.1 (5.4)	15.7 (5.4)
Study 3: Subthalamic nucleus connectivity in pathological drinking and exploratory machine learning analysis	HV for BD	16/16	24.1 (3.4)	116.3 (6.3)	4.5 (4.9)	4.2 (4.6)
	HV for AUD	17/17	41.4 (12.5)	117.6 (7.3)	6.3 (7.9)	3.9 (2.2)
	BD	18/14	22.1 (3.3)	116.5 (5.3)	7.13 (5.4)	15.7 (5.4)
	AUD	21/15	40.6 (12.1)	113.5 (6.2)	12.1 (9.1)	
Subthalamic nucleus connectivity in social drinkers as a function of alcohol use severity	HV	14/24	35.1 (15.1)	115.8 (12.4)	3.8 (5.0)	4.3 (3.5)
	BD	18/14	22.1 (3.3)	116.5 (5.3)	7.1 (5.4)	15.7 (5.4)

AUD, alcohol use disorders; AUDIT, Alcohol Use Disorders Test; BD, binge drinkers; BDI, Beck Depression Inventory; HV, healthy volunteers; M/F, male/female; SD, standard deviation; STN, subthalamic nucleus.

Supplemental References

1. Beck AT, Erbaugh J, Ward CH, Mock J, Mendelsohn M (1961): An Inventory for Measuring Depression. *Arch Gen Psychiat*. 4:561-7.
2. (NIAAA) NIoAAaA (2004) NIAAA council approves definition of binge drinking. NIAAA Newsletter 3.
3. Association AP (2000) Diagnostic and statistical manual of mental disorders (4th Ed., text rev). Washington, D.C.: American Psychiatric Association.
4. Aron AR, Fletcher PC, Bullmore ET, Sahakian BJ, Robbins TW (2003) Stop-signal inhibition disrupted by damage to right inferior frontal gyrus in humans. Nature neuroscience 6:115-116.
5. Kundu P, Inati SJ, Evans JW, Luh WM, Bandettini PA (2012) Differentiating BOLD and non-BOLD signals in fMRI time series using multi-echo EPI. NeuroImage 60:1759-1770.
6. Kundu P, Brenowitz ND, Voon V, Worbe Y, Vertes PE, Inati SJ, Saad ZS, Bandettini PA, Bullmore ET (2013) Integrated strategy for improving functional connectivity mapping using multiecho fMRI. Proceedings of the National Academy of Sciences of the United States of America 110:16187-16192.
7. Schrouff J, Rosa MJ, Rondina JM, Marquand AF, Chu C, Ashburner J, Phillips C, Richiardi J, Mourao-Miranda J (2013) PRoNTo: pattern recognition for neuroimaging toolbox. Neuroinformatics 11:319-337.
8. Orru G, Pettersson-Yeo W, Marquand AF, Sartori G, Mechelli A (2012) Using Support Vector Machine to identify imaging biomarkers of neurological and psychiatric disease: A critical review. Neuroscience and biobehavioral reviews 36:1140-1152.
9. Maldjian JA, Laurienti PJ, Kraft RA, Burdette JH (2003) An automated method for neuroanatomic and cytoarchitectonic atlas-based interrogation of fMRI data sets. NeuroImage 19:1233-1239.
10. Aron AR, Behrens TE, Smith S, Frank MJ, Poldrack RA (2007) Triangulating a cognitive control network using diffusion-weighted magnetic resonance imaging (MRI) and functional MRI. The Journal of neuroscience 27:3743-3752.
11. Weiskopf N, Suckling J, Williams G, Correia MM, Inkster B, Tait R, Ooi C, Bullmore ET, Lutti A (2013) Quantitative multi-parameter mapping of R1, PD(*), MT, and R2(*) at 3T: a multi-center validation. Frontiers in neuroscience 7:95.
12. Murray GK, Corlett PR, Clark L, Pessiglione M, Blackwell AD, Honey G, Jones PB, Bullmore ET, Robbins TW, Fletcher PC (2008) Substantia nigra/ventral tegmental reward prediction error disruption in psychosis. Mol Psychiatry 13:239, 267-276.
13. Martinez D, Slifstein M, Broft A, Mawlawi O, Hwang DR, Huang Y, Cooper T, Kegeles L, Zarahn E, Abi-Dargham A, Haber SN, Laruelle M (2003) Imaging human mesolimbic dopamine transmission with positron emission tomography. Part II: amphetamine-induced dopamine release in the functional subdivisions of the striatum. Journal of cerebral blood flow and metabolism 23:285-300.
14. Tzourio-Mazoyer N, Landeau B, Papathanassiou D, Crivello F, Etard O, Delcroix N, Mazoyer B, Joliot M (2002) Automated anatomical labeling of activations in SPM using a macroscopic anatomical parcellation of the MNI MRI single-subject brain. NeuroImage 15:273-289.
15. Kim JH, Lee JM, Jo HJ, Kim SH, Lee JH, Kim ST, Seo SW, Cox RW, Na DL, Kim SI, Saad ZS (2010) Defining functional SMA and pre-SMA subregions in human MFC using resting state fMRI: functional connectivity-based parcellation method. NeuroImage 49:2375-2386.

16. Desikan RS, Segonne F, Fischl B, Quinn BT, Dickerson BC, Blacker D, Buckner RL, Dale AM, Maguire RP, Hyman BT, Albert MS, Killiany RJ (2006) An automated labeling system for subdividing the human cerebral cortex on MRI scans into gyral based regions of interest. NeuroImage 31:968-980.
17. Cox SR, Ferguson KJ, Royle NA, Shenkin SD, MacPherson SE, MacLullich AM, Deary IJ, Wardlaw JM (2014) A systematic review of brain frontal lobe parcellation techniques in magnetic resonance imaging. Brain structure & function 219:1-22.
18. Johnson-Frey SH, Maloof FR, Newman-Norlund R, Farrer C, Inati S, Grafton ST (2003) Actions or hand-object interactions? Human inferior frontal cortex and action observation. Neuron 39:1053-1058.
